# Financial inclusion and intimate partner violence: What does the evidence suggest?

**DOI:** 10.1371/journal.pone.0223721

**Published:** 2019-10-16

**Authors:** Lotus McDougal, Jeni Klugman, Nabamallika Dehingia, Amruta Trivedi, Anita Raj

**Affiliations:** 1 Center on Gender Equity and Heath, Department of Medicine, School of Medicine, University of California San Diego, San Diego, CA, United States of America; 2 Georgetown Institute for Women, Peace and Security, Georgetown University, Washington, DC, United States of America; 3 Department of Education Studies, University of California San Diego, San Diego, CA, United States of America; Washington University in St. Louis, UNITED STATES

## Abstract

Financial inclusion is an area of growing global interest in women’s empowerment policy and programming. While increased economic autonomy may be expected to reduce the prevalence of intimate partner violence, the mechanisms and contexts through which this relationship manifests are not well understood. This analysis aims to assess the relationship between women’s financial inclusion and recent intimate partner violence using nationally-representative data from 112 countries worldwide. Levels of both financial inclusion and recent intimate partner violence varied substantially across countries (ranging from 2–100%, and 1–46%, respectively), and across regions. In multivariate global analyses, increased levels of women’s financial inclusion were associated with lower levels of recent intimate partner violence after accounting for asset-based enablers of economic autonomy and gender norms; this relationship was lost upon the inclusion of measures of national context (i.e., development and fragility). These results underscore that the relationship between financial inclusion and recent intimate partner violence is complex, follows many pathways, and is affected by context. In low and middle income countries, asset-based enablers of economic autonomy, gender norms and national context explained much of the relationship between financial inclusion and recent intimate partner violence. In those low and middle income countries with high levels of controlling behavior by male spouses, financial inclusion was associated with higher levels of recent intimate partner violence. These findings further suggest that initiatives that aim to prevent intimate partner violence by way of increased economic autonomy may be ineffective in the absence of broader social change and support, and indeed, as seen in countries with higher levels of men’s controlling behavior, backlash may increase the risk of violence. Efforts to improve women’s financial inclusion need to recognize that its relationship with intimate partner violence is complex, and that it requires an enabling environment supportive of women’s rights and autonomy.

## Introduction

Nearly one in five women globally (19%) have experienced sexual or physical violence from an intimate partner in the past year, with wide variations in prevalence across countries [1]. Beyond the violation of human rights, intimate partner violence (IPV) has substantial health implications for women and their children, including increased risk of abortion, premature birth, low birth weight, sexually transmitted infections, depression and substance abuse, as well as death [2]. There has thus been an increasing focus on modalities of prevention, including ways in which women’s economic autonomy may reduce the risk of IPV.

Both preventing IPV and expanding women’s economic autonomy have become prominent features in the global goals and policy agenda [3, 4]. While these two phenomena address distinct areas of women’s lives, there is increasing evidence that they may be linked. Possible mechanisms through which increased economic autonomy may reduce IPV include lessening financial stress on the household, reducing women’s financial dependence on men and enabling women to leave relationships if they so choose [5–9]. The level and duration of violence reduction seen in interventions designed to boost women’s economic autonomy appears to be context- and population-specific, however, and in some cases, has been associated with increased rather than decreased IPV risk [5, 10, 11].

There is evidence suggesting that the ability to exit an abusive relationship, which may be facilitated by augmented economic autonomy, can deter further IPV. For example, state level family law reforms facilitating divorce in the United States reduced the risk of marital violence [12]. In contrast, studies from Ghana and Bangladesh suggest that microfinance loans and cash transfers may increase IPV due to disagreement over use and control of the additional income [11, 13]. Contexts where gender roles are in the process of shifting, and thus where gender power dynamics are in flux, may be more risky for women. This variability is echoed in research on a broader array of economic empowerment indicators, including women’s employment and control over resources or assets [5, 14]. More recent multi-country studies have explored the relationship between economic measures of women’s status and IPV, suggesting that restrictions on legal rights and employment are associated with higher levels of IPV [15, 16].

Financial inclusion, encompassing both access to and use of appropriate financial services, is emerging as a focal area of efforts to increase women’s economic autonomy [3]. Financial inclusion has been identified as a key enabler for many of the Sustainable Development Goals, including the goal of achieving gender equality and enhancing women’s empowerment [17]. Levels of women’s financial inclusion are highly varied across countries, from a low of 13% financial account ownership among women in South Sudan, to effectively universal in many high income economies [3]. Financial inclusion of women has shown promise as a way to increase women’ economic empowerment, including indication of increased savings and financial resilience, as well as diversification of food purchases, in diverse settings including Kenya, Nepal and Niger [18–21].

**Fig 1:**
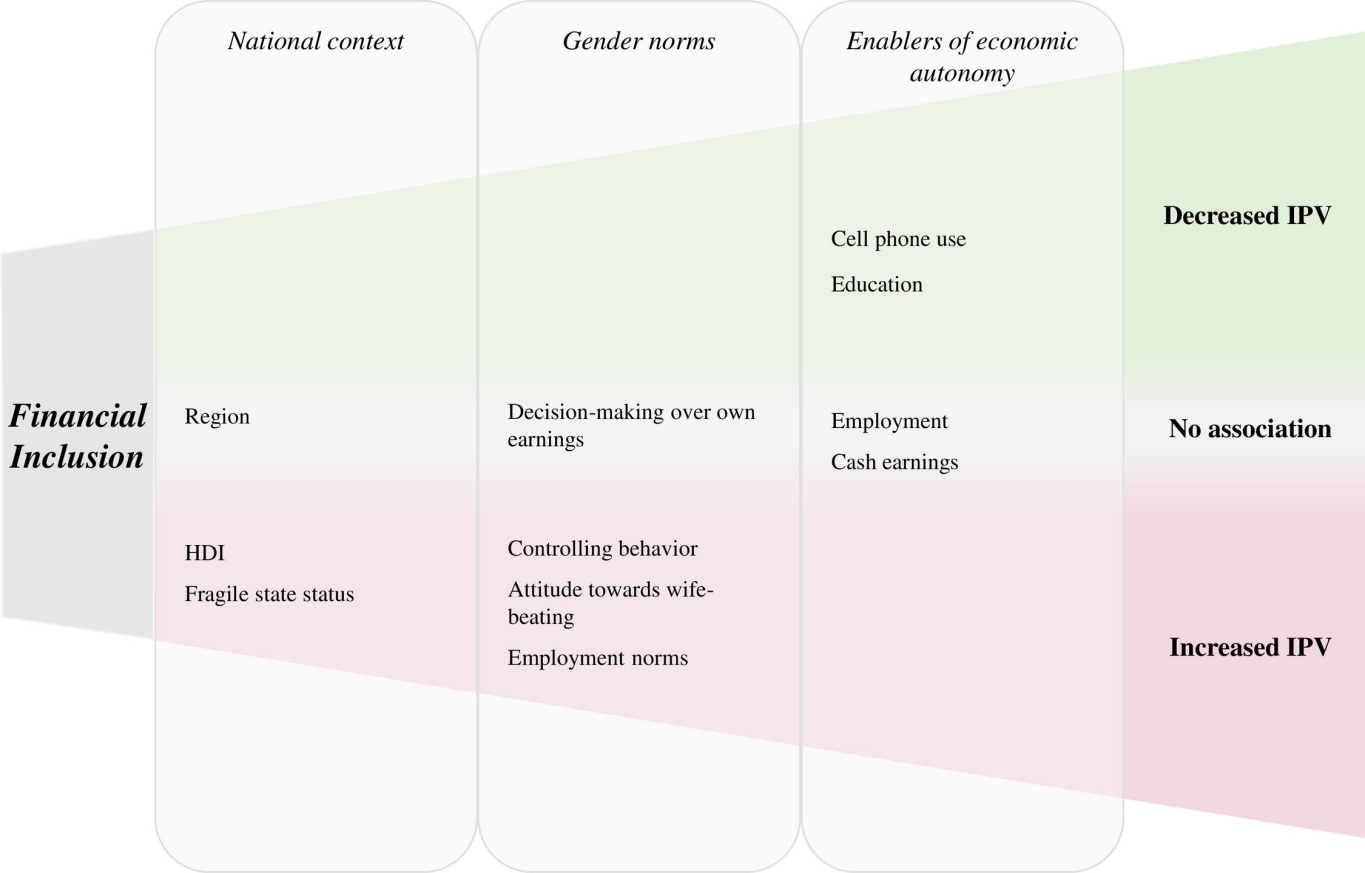
Conceptual model outlining factors influencing the relationship between financial inclusion and intimate partner violence.

To date, there is limited research exploring the relationship between financial inclusion and IPV, though evidence from India suggests that bank account ownership is associated with reduced risk of IPV [22]. Women’s access to more resources may be expected to increase their autonomy, yet might also lead to backlash and a heightened risk of violence if men seek to maintain power differentials. A summary of factors influencing potential pathways connecting financial inclusion and IPV is outlined in Fig 1.

The majority of theoretical models examining factors affecting IPV have used the ecological model to look broadly at risks from an individual up to a societal lens [23–25]. This analysis differs from these approaches in that nationally-representative measures are used to assess cross-national associations at the macro level. Our conceptual model therefore focuses on explicating factors important to the relationship between women’s financial inclusion and recent IPV at the societal (national context) and community (gender norms, enablers of economic autonomy) levels.

This paper thus aims to address the gap in knowledge connecting the role of financial inclusion and risk of IPV. Specifically, we assess, for a large and diverse sample of countries, whether women’s financial inclusion–defined as having an account (alone or jointly) at a bank or another type of financial institution or personally using a mobile money service in the past 12 months—is associated with lower levels of recent IPV, accounting for key contextual, normative and enabling factors.

## Methods

### Study design and sample

This study is an ecological analysis of the relationship between financial inclusion and IPV using publicly available, cross-sectional, country-level data from multiple sources (see Table 1). We drew data on recent IPV from the UN Women Global Database on Violence against Women where available; countries missing IPV data in this database were added where feasible using nationally-representative individual sources outlined in S1 Table [26]. The UN Women Global Database on Violence against Women includes IPV prevalence data from multiple sources including population surveys, violence-focused surveys and national statistics offices. Countries with recent IPV data available for 2005 or later were included in this study.

**Table 1:** Measures and data sources for all variables.

Measure	Definition	Year	Countries (total = 112)	Data Sources
Recent IPV	Of all ever-partnered women and girls aged 15–49, percent who reported physical and/or sexual violence from an intimate partner in the previous 12 months	2005–2018	112	UN Women Global Database on Violence against Women, individual sources as noted in S1 Table (most recent year available)
Financial inclusion	Of all women and girls aged 15+, percent who reported having an account (sole or joint) at a bank or another type of financial institution or personally using a mobile money service in the previous 12 months.	2011, 2014, 2017	112	Global Findex database, World Bank (closest year match to recent IPV year)
Financial inclusion gender gap	Gap between male and female financial inclusion prevalences. Positive values indicate greater male financial inclusion, negative values indicate greater female inclusion.	2011, 2014, 2017	112	Global Findex database, World Bank (closest year match to recent IPV year)
***Asset-based enablers of economic autonomy***				
Employment	Of all women aged 25 or older, percent who are employed.	2016	112	ILOSTAT
Cash earnings	Of all currently married women and girls aged 15–49 who were employed in the past 12 months, percent who received cash for their earnings	2005–2018	52	DHS StatCompiler (closest year match to recent IPV year)
Cell phone use	Of women aged 15 or older, percent who report having a mobile phone used to make and receive personal calls.	2015	112	2015 Gallup World Poll, via the Women, Peace and Security Index
Education	Average years of education among women aged 15 or older.	2005, 2010	99	Barro-Lee estimates (closest year match to recent IPV year)
***Gender norms***				
Inequitable employment norms	Of men aged 15 or older, percent who disagreed that “*It is perfectly acceptable for any woman in your family to have a paid job outside the home IF SHE WANTS ONE*.”	2015	101	2015 Gallup World Poll, via Gallup/ ILO *Towards a better future for women and work*: *Voices of women and men* report
Decision-making over own earnings	Of all currently married women and girls aged 15–49 who were employed in the past 12 months and received cash for their earnings, percent who were involved in decision-making over how those cash earnings would be used.	2005–2018	52	DHS StatCompiler (closest year match to recent IPV year)
Controlling behavior	Of all ever-partnered women and girls aged 15–49, percent who reported their partner exhibiting at least one of the following: being jealous or angry if she talks to other men, frequently accusing her of being unfaithful, not permitting her to meet her female friends, trying to limit her contact with her family, insisting on knowing where she is at all times, not trusting her with any money.	2005–2018	47	DHS StatCompiler (closest year match to recent IPV year)
Wife-beating justified	Of all women and girls aged 15–49, percent who agree that wife beating is justified for at least one of the following: if she burns the food, if she argues with him, if she goes out without telling him, if she neglects the children, if she refuses to have sex with him.	2005–2018	53	DHS StatCompiler (closest year match to recent IPV year)
***National context***				
HDI	Geometric mean of normalized indices for three dimensions: long and healthy life (life expectancy index), knowledge (education index) and a decent standard of living (GNI index).	2005–2017	112	United Nations Development Programme (closest year match to recent IPV year)
Fragile state	Countries or territories with 1) a harmonized Country Policy and Institutional Assessment country rating ≤ 3.2, and/or 2)the presence of a UN and/or regional peace-keeping or political and peace-building mission within the last three years.	2018	112	World Bank Harmonized List of Fragile Situations, FY18List
Region	Sustainable Development Goal region, based on the United Nations’ M49 standard groupings. High income countries, as defined by the World Bank’s income groupings, are categorized as a separate region.	2018	112	United Nations Statistics Division and World Bank

Financial inclusion data was drawn from the World Bank’s Global Findex database, which compiles data collected from nationally representative triennial surveys (in 2011, 2014 and 2017) of individual financial behavior collected by Gallup, Inc.

Employment rates come from ILOSTAT [27], in which ILO models employment-to-population ratios from using data from labor force surveys, household surveys and population censes; all estimates used are from 2016.

Employment norms and cell phone use were taken from the 2015 Gallup World Poll, via reports from Gallup/ILO and the Women, Peace and Security Index [28, 29]. Cash earnings, decision-making over own earnings, controlling behavior and justification for wife-beating were taken from DHS StatCompiler for the year most closely matched to the year of recent IPV data (2005–2018) [30].

Human Development Index (HDI) values for the year most closely matched to the year of recent IPV data for (2005–2017) were taken from the United Nations Development Programme [31]. The HDI is comprised of indicators on life expectancy, actual and expected education, and GNI per capita [32]. Fragile states were identified via World Bank categorizations (2018) [33, 34]. Regions were defined according to the United Nation’s M49 groupings, with the exception of high income countries (as defined by the World Bank’s income groupings), which were categorized separately [35, 36]. Female education (mean years of schooling) was sourced from Barro-Lee estimates (2005 and 2010) for the year most closely matched with recent IPV data [37].

Data were extracted in March 2019.

The sample was limited to the 112 countries with data on recent (past 12-month) IPV, as well as financial inclusion, of which 33 are defined by the World Bank as high income (see S1 Table) [38].

### Measures

Measure definitions and sources are shown in Table 1. We defined the primary dependent variable, recent IPV, as the percent of ever-married women reporting any physical and/or sexual violence in the preceding 12 months. Physical violence includes being pushed or shaken, having something thrown at you, having an arm twisted or hair pulled, being slapped, being punched with a fist or something else that could hurt the respondent, being kicked, dragged or beaten up, trying to intentionally choke or burn, or being threatened or attacked with a knife, gun or other weapon. Sexual violence includes being physically forced to have sex or other unwanted sexual acts, or being forced with threats or in other ways to perform unwanted sexual acts. These definitions are in accordance with globally accepted measures of gender-based violence [2].

Financial inclusion is defined as the percent of women aged 15 or older who reported having an independent or joint account at a bank or another type of financial institution, or personally using a mobile money service in the previous 12 months.

We grouped the covariates into three domains: (1) asset-based enablers of economic autonomy, (2) gender norms related to women’s lower status and control and (3) national context. Variables in (1) included paid employment, cash earnings, cell phone use and education. Variables in (2) included inequitable employment norms, decision-making over own earnings, controlling behavior and justification for wife-beating. Variables in (3) included HDI, fragile state status and region.

All measures except HDI, fragile state status and region are derived from individual-level data and expressed as national prevalence estimates.

### Analysis

Descriptive analyses included frequency statistics for all variables, overall and by region.

We first assessed the relationship between independent variables and recent IPV using bivariate and multivariate fractional logit generalized linear models, to account for the bounded nature of the outcome (recent IPV). Full-sample regressions were modelled using sequential variable inclusion by measure grouping (asset-based enablers of economic autonomy, gender norms, and national context).

We also modelled limited-sample regressions excluding high-income countries in order to include variables with limited geographic availability (cash earnings, decision-making control over own earnings, controlling behavior, and justification for wife-beating). All regressions included robust standard errors clustered at the regional level, and multivariate models adjusted for IPV year fixed effects.

All datasets used contained only country-level, de-identified data.

## Results

**Fig 2:**
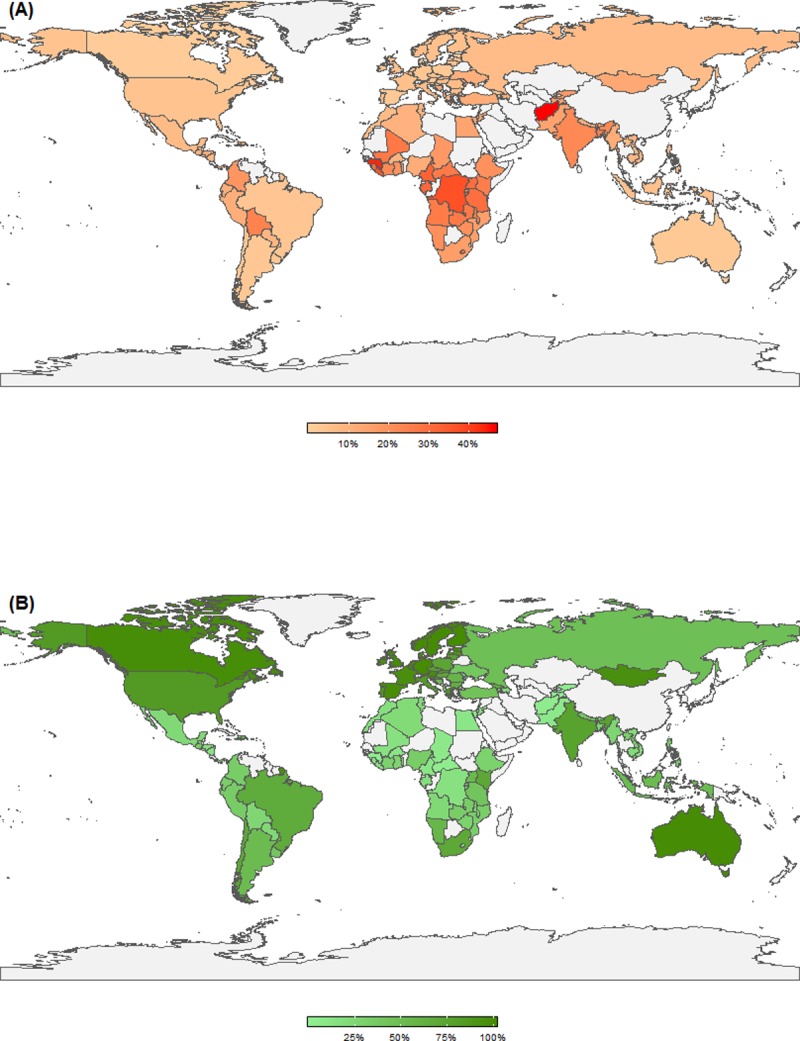
Prevalence of recent intimate partner violence (A) and women’s financial inclusion (B) as reported by women across assessed countries (n = 112).

Globally, the median prevalence of recent physical and/or sexual violence in assessed countries was 9% (Table 2), ranging from less than 1% in Singapore to 46% in Afghanistan. Countries in Sub-Saharan Africa, and Central and Southern Asia tended to have higher prevalence of recent IPV than Northern America and Europe (Fig 2).

**Table 2:** Descriptive statistics of recent intimate partner violence, financial inclusion and related covariates.

	Global (n = 112)	High Income Countries (n = 33)	Central Asia and Southern Asia (n = 10)	Eastern Asia and South-Eastern Asia (n = 7)	Latin America and the Caribbean (n = 17)	Northern America and Europe (n = 4)	Sub-Saharan Africa (n = 30)	Western Asia and Northern Africa (n = 11)
	Median (range)	Median (range)	Median (range)	Median (range)	Median (range)	Median (range)	Median (range)	Median (range)
Recent IPV (%)	9.2	4.0	14.9	7.7	8. 5	8.1	24.9	9.4
	(0.9–46.1)	(0.9–6.0)	(6.0–46.1)	(4.9–12.7)	(2.7–24.2)	(6.0–11.5)	(4.9–43.6)	(1.1–14.7)
Financial inclusion (%)	38.5	95.6	26.8	31.9	33.2	48.0	24.7	20.7
	(2.1–100.0)	(50.9–100.0)	(2.1–76.6)	(18.9–95.0)	(10.1–71.3)	(17.2–63.2)	(2.9–87.1)	(9.3–63.6)
Financial inclusion gender gap (%)	4.9	0.1	9.8	-4.1	7.3	3.3	6.7	14.8
	(-13.5–27.6)	(-5.7–14.6)	(-0.4–27.6)	(-13.5–5.1)	(-3.8–16.5)	(-0.4–8.3)	(-1.6–20.3)	(-5.1–25.7)
***Asset-based enablers of economic autonomy***								
Female employment (%)	54.0	52.7	42.5	58.1	54.8	45.8	69.8	23.2
	(13.5–92.7)	(35.5–62.2)	(24.0–83.0)	(49.9–82.2)	(42.8–70.4)	(42.9–47.6)	(39.9–92.7)	(13.5–65.6)
Cash earnings (%)	70.4	-	72.6	83.0	85.8	88.2	54.4	82.4
	(16.9–95.9)		(35.9–88.0)	(68.9–86.7)	(72.0–92.0)	(80.5–95.9)	(16.9–94.7)	(65.3–84.1)
Cell phone use (%)	82.3	93.2	76.9	69.5	79.9	85.0	60.6	86.2
	(25.9–100.0)	(76.6–99.5)	(32.6–95.5)	(60.4–97.5)	(62.1–94.0)	(79.9–89.1)	(25.9–86.9)	(79.2–92.4)
Female education (years)	8.6	11.0	4.8	6.8	8.1	10.9	5.2	6.6
	(1.4–13.2)	(7.4–13.2)	(2.0–11.4)	(4.0–9.5)	(3.7–10.1)	(10.4–11.2)	(1.4–9.6)	(4.1–10.6)
***Gender norms***								
Inequitable employment norms (%)	11.0	3.0	28.0	17.0	9.5	9.0	15.0	26.0
	(0.0–73.0)	(0.0–26.0)	(5.0–73.0)	(8.0–37.0)	(4.0–22.0)	(6.0–11.0)	(6.0–30.0)	(6.0–48.0)
Decision-making over own earnings (%)	91.0	-	84.7	95.2	96.3	97.8	89.1	91.9
	(65.0–98.4)		(74.2–94.3)	(91.7–98.2)	(83.0–98.4)	(97.4–98.1)	(65.0–96.7)	(90.1–94.5)
Controlling behavior (%)	65.2	-	58.6	29.1	64.9	67.4	65.1	77.2
	(27.2–86.4)		(28.1–81.9)	(27.2–34.1)	(51.8–71.8)	(65.9–68.9)	(35.4–86.4)	(49.4–84.6)
Wife-beating justified (%)	36.6	-	41.1	42.2	11.0	12.2	45.0	22.6
	(2.3–92.1)		(28.5–80.2)	(12.9–51.2)	(2.3–16.7)	(3.6–20.8)	(5.5–92.1)	(6.8–49.0)
***National context***								
HDI	0.71	0.89	0.61	0.65	0.71	0.76	0.50	0.73
	(0.35–0.94)	(0.78–0.94)	(0.49–0.79)	(0.57–0.74)	(0.49–0.84)	(0.68–0.80)	(0.35–0.79)	(0.61–0.78)
Fragile state^1^								
No	96 (85.7)	33 (100.0)	9 (90.0)	6 (85.7)	16 (94.1)	4 (100.0)	18 (60.0)	10 (90.9)
Yes	16 (14.3)	0 (0.0)	1 (10.0)	1 (14.3)	1 (5.9)	0 (0.0)	12 (40.0)	1 (9.1)

^1^ N (%).

The median prevalence of financial inclusion was 39%, with near saturation in high-income countries, and only 21% median prevalence in Western Asia and Northern Africa. Across the global sample, the prevalence of women’s employment was 54%, ranging from a regional high of 70% in Sub-Saharan Africa to 23% in Western Asia and Northern Africa. Cell phone use was high in the total sample (82%) and women had a median of nearly nine years of education.

Among indicators of gender norms, inequitable employment norms were highest in Central Asia and Southern Asia (28%) and West Asia and Northern Africa (26%).Most countries had high levels of female participation in decision making regarding their own earnings (global median 91%). However, more than one-half of women (65%) across assessed countries reported controlling behavior by partners (a measure indicative of spousal power imbalances). The median level of beliefs that wife beating was justifiable was 37% and of the sampled countries.

HDI was highest among high income countries (median of 0.89) (Table 2). Sub-Saharan Africa had both the lowest HDI of any region in the sample (median of 0.50), as well as the vast majority of states classified as fragile (12 of 16).

**Fig 3:**
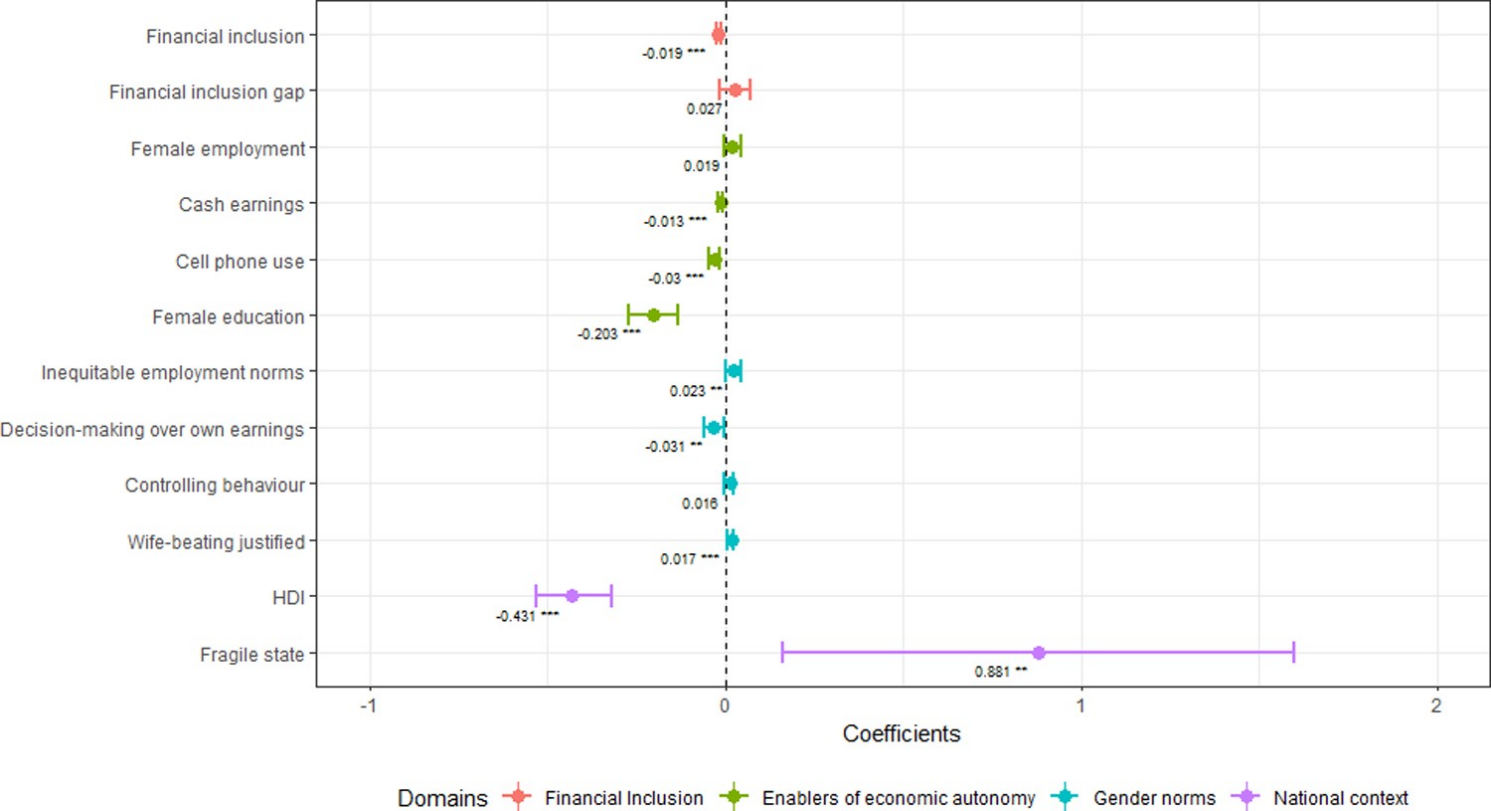
Bivariate logit generalized linear models assessing the association between recent IPV and individual independent variables. *p<0.10; **p<0.05; ***p<0.001. Note: Each line represents coefficients and confidence intervals for individual regression models. HDI coefficient represents a 10% increase in HDI.

In bivariate analyses, women’s financial inclusion was negatively associated with recent IPV; for every 10% increase in financial inclusion, there was a 2% decrease in recent IPV (Fig 3). Inequitable employment norms, justification of wife-beating and fragile state status were all positively associated with IPV; whereas cash earnings, cell phone use, female education, women’s decision-making over their own earnings and HDI were negatively associated with recent IPV.

In multivariate analyses, there was a significant, negative association between financial inclusion and recent IPV in models adjusted for measures of economic autonomy and gender norms (Table 3). Model 3 (adjusted for both asset-based enablers of economic autonomy and gender norms) had the best overall fit, and also demonstrated an association between increased female education and lower levels of IPV. However, upon inclusion of measures of national context, the statistical association between financial inclusion and recent IPV was lost. Model 4 necessarily excluded female education due to its high correlation with HDI (r = 0.90; p-value<0.01; mean years of education for the general population is a component of the HDI [32]). While fragile states tended to have higher levels of IPV in bivariate analyses, there was no association in multivariate analyses. Women’s employment and inequitable employment norms were not significantly associated with recent IPV in any model.

**Table 3:** Associations of financial inclusion and covariates with recent intimate partner violence.

	Model 1	Model 2: Model 1 + enablers	Model 3: Model 2 + gender norms	Model 4: Model 3 + national context
Financial inclusion	-0.020	-0.010	-0.009	-0.002
	(<0.01)	(<0.01)	(0.01)	(0.47)
Financial inclusion gender gap	-0.002	-0.005	-0.009	-0.002
	(0.86)	(0.73)	(0.59)	(0.81)
**Asset-based enablers of economic autonomy**				
Female employment		0.004	0.010	0.0001
		(0.42)	(0.13)	(0.97)
Cell phone use		-0.003	-0.003	0.006
		(0.50)	(0.53)	(0.04)
Female education		-0.117	-0.109	-
		(<0.01)	(<0.01)	-
**Gender norms**				
Inequitable employment norms			0.009	0.005
			(0.25)	(0.32)
**National context**				
HDI (10% increase)				-0.474
				(<0.01)
Fragile state				
No				Reference
Yes				-0.133 (0.43)
Observations	112	99	91	101
*AIC*	66.12	61.56	57.34	62.45

Numbers presented are coefficients from fractional logit models, with p-values in parentheses. All models contain IPV year fixed effects. Robust standard errors are clustered on regions.

Recognizing that high-income countries are distinct from others in our sample, characterized by lower levels of IPV and close to universal financial inclusion (see Table 2), we performed an exploratory set of analyses restricted to low and middle income countries. In these reduced models excluding high-income nations and adjusting for asset-based enablers of economic autonomy and gender norms, the relationship between women’s financial inclusion and recent IPV was not statistically significant (Table 4). However, when we additionally controlled for cash earnings, decision-making over own earnings, controlling behavior and justification for wife beating (measures available only for low and middle income countries in this sample), higher levels of women’s financial inclusion were associated with higher levels of recent IPV, even controlling for national context.

**Table 4:** Associations of financial inclusion and covariates with recent intimate partner violence among low and middle income countries.

	Model 1	Model 2: Model 1 + enablers	Model 3: Model 2 + gender norms	Model 4: Model 3 + national context	Model 5: Model 4 + additional measures
Financial inclusion	-0.014	-0.003	-0.001	0.002	0.011
	(<0.01)	(0.17)	(0.76)	(0.12)	(<0.01)
Financial inclusion gender gap	-0.005	-0.008	-0.010	-0.004	-0.008
	(0.72)	(0.66)	(0.57)	(0.75)	(0.34)
**Asset-based enablers of economic autonomy**					
Female employment		0.003	0.010	0.001	0.003
		(0.51)	(0.14)	(0.84)	(0.46)
Cash earnings					0.003
					(0.74)
Cell phone use		-0.008	-0.007	0.002	-0.005
		(0.08)	(0.16)	(0.52)	(0.05)
Female education		-0.094	-0.087	-	-
		(0.01)	(<0.01)	-	-
**Gender norms**					
Inequitable employment norms			0.010	0.005	0.007
			(0.32)	(0.41)	(0.46)
Decision-making over own earnings					-0.013
					(0.27)
Controlling behavior					0.024
					(<0.01)
Wife-beating justified					0.011
					(<0.01)
**National context**					
HDI (10% increase)				-0.411	-0.081
				(<0.01)	(0.72)
Fragile state					
No				Reference	Reference
Yes				-0.039 (0.81)	0.020 (0.92)
Observations	79	66	61	71	40
*AIC*	57.26	52.73	49.45	52.76	35.80

Numbers presented are coefficients from fractional logit models, with p-values in parentheses. All models contain outcome year fixed effects. Robust standard errors are clustered on regions.

**Fig 4:**
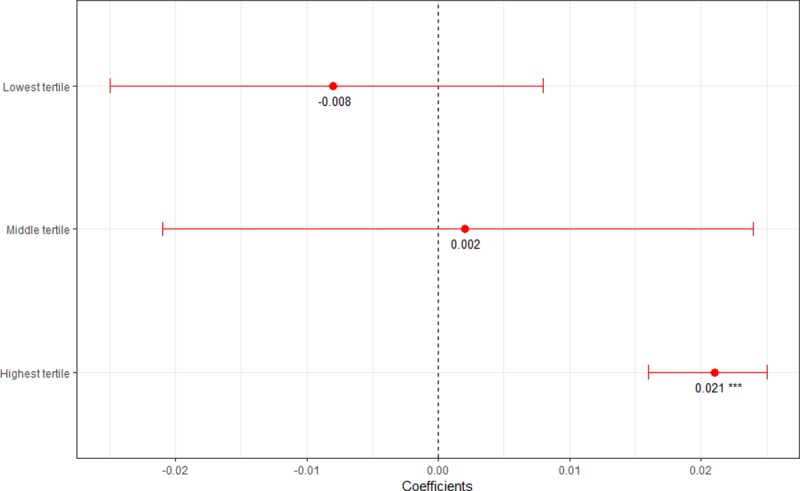
Association between women’s financial inclusion and recent IPV stratified by levels of controlling behavior. *p<0.10; **p<0.05; ***p<0.001. Note: Fig coefficients show associations between women’s financial inclusion and recent IPV from fractional logit models, adjusting for controlling behavior and outcome year fixed effects, with robust standard errors clustered on regions. Tertiles are defined by national prevalence of controlling behavior, delineated as follows: 27.2%-56.7% (lowest), 60.5%-68.9% (middle) and 71.4%-86.4% (highest). Countries in the lowest tertile of controlling behavior include Armenia, Burundi, Cambodia, Ethiopia, Guatemala, India, Mali, Mozambique, Myanmar, Namibia, Nepal, Pakistan, Philippines, Rwanda and South Africa. Countries in the middle tertile of controlling behavior include Afghanistan, Angola, Burkina Faso, Chad, Comoros, Côte d’Ivoire, Dominican Republic, Ghana, Honduras, Kenya, Nigeria, Peru, Republic of Moldova, Togo, Ukraine and Zimbabwe. Countries in the highest tertile of controlling behavior include Azerbaijan, Cameroon, Democratic Republic of the Congo, Egypt, Gabon, Haiti, Jordan, Kyrgyzstan, Liberia, Malawi, Sierra Leone, Tajikistan, Uganda, United Republic of Tanzania and Zambia.

Exploratory analysis of the full model (Table 4, Model 5) revealed that controlling behavior was the most important measure in establishing this significance (results not shown). The relationship between women’s financial inclusion and recent IPV was then plotted by tertile of controlling behavior (Fig 4).

## Discussion

While the prevalence of both financial inclusion and recent IPV vary widely across countries, in general, higher levels of women’s financial inclusion were associated with lower levels of recent IPV, even after accounting for asset-based enablers of economic autonomy and gender norms. In the context of rising women's financial inclusion (from 47% worldwide in 2011 to 65% in 2017 [3]), this is an encouraging finding. This result may be at least partially explained by other research suggesting that increased financial inclusion may enable greater autonomy and exit options [5, 12]. It is also in line with longitudinal data from rural India in which both bank account ownership and joint control over husbands’ income reduced IPV risk [22]. The relationship between women’s financial inclusion and recent IPV lost its statistical association, however, when controls for national context (HDI and fragile state status) were introduced. This suggests that overall levels of development, as proxied by HDI, play a key role in explaining levels of recent IPV when looking at a countries across all income levels.

In models restricted to low and middle income countries, the relationship between levels of women’s financial inclusion and recent IPV, adjusting for the gender gap in financial inclusion, was similar to that seen in the global sample. However this association became non-significant when additional controls were introduced for asset-based enablers of economic autonomy, gender norms and national context, suggesting that at present, these contextual factors explain much of the relationship originally seen between financial inclusion and recent IPV in low and middle income countries. The association between HDI and recent IPV was lost upon accounting for cash earnings and gender norms such as controlling behavior and justification of wife-beating, which is in line with previous research, and emphasizes differences in correlates of IPV across contexts [15].

One interesting and cautionary result is the reversal of the relationship between women’s financial inclusion and recent IPV within low and middle income countries in models that included additional measures of asset-based enablers of economic autonomy and gender norms. Upon adjusting for cash earnings, decision-making over earnings, controlling behavior, and the justification of wife-beating, low and middle income countries with higher levels of financial inclusion tended to have higher levels of IPV. The significance of this result, however, was conditioned on levels of controlling behavior; the positive association between increased women’s financial inclusion and increased recent IPV was only significant in countries where more than 70% of women reported that their spouses exhibited at least one controlling behavior. These countries with higher levels of controlling behavior also tended to be contexts with higher levels of justification of wife beating and higher levels of women’s employment, but also lower levels of cash earnings and more inequitable employment norms (S2 Table). These are circumstances where women have increased financial inclusion in a context of high spousal control and generally compromised gender norms, which may be aggravating gendered power struggles, a situation which has been known to result in increased IPV [14]. This finding may also be in part explained by other studies indicating that expanding economic opportunities for women are most likely to reduce IPV when women’s social status is also augmented, and that that social status exists within a confluence of other factors perpetuating gender norms [39, 40]. Indeed, recent research from Ethiopia has found that the different dimensions of women’s empowerment, particularly economic empowerment, are largely distinct from one another [41]. This suggests that unidimensional empowerment initiatives may not necessarily have spillover effects into other dimensions and that multipronged efforts are needed to effect more broad-reaching change [23, 42–44].

The issue of male control is also of relevance when considering the measurement of women’s financial inclusion. The measure used in this study assesses account ownership (bank or other financial institution) or mobile money service use within the past year among women and girls aged 15 or older; this is a gender-specific version of the indicator used to track progress on Sustainable Development Goal 8.10.2 [45]. The phrasing of the Global Findex Questionnaire does not allow for disaggregation of sole vs. joint accounts: “Do you, either by yourself or together with someone else, currently have an account at a bank or another type of formal financial institution?” [46]. When examining financial inclusion in the context of autonomy, empowerment and risk, this is a key distinction. Women with joint accounts may have more explicit and/or implicit restrictions and limitations on their use of that account than women with sole accounts. Conversely, it is plausible that in some contexts, joint ownership may indicate higher levels of trust and lower levels of control within a relationship [47, 48]. These dynamics are poorly understood and understudied, and merit additional research to better understand whether this conflation of ‘joint’ and ‘sole’ accounts is masking important differences in the measurement of women’s financial inclusion.

While fragile state status was associated with increased risk of recent IPV in bivariate analyses, it did not emerge as significant in multivariate analyses. There are several possible explanations for this. The prevalence of recent IPV in fragile states had much wider variability than was seen in non-fragile states (mean standard errors of 2.8 and 0.9, respectively). There was only modest variability in financial inclusion in fragile states (ranging only from 3%-33%).This contrast is clear in Afghanistan, which has the highest level of recent IPV (46%) seen across assessed countries, and one of the lowest levels of women’s financial inclusion (4%), vs. Chad, which has 18% recent IPV and 8% women’s financial inclusion. This relationship may have been further compromised in multivariate models by the fact that only 16 of 112 countries were considered fragile states, and sample size was thus limited for this measure. Finally, while previous research has indicated that the normalization of violence in society more broadly may be associated with higher levels of violence against women in the home [49], fragile state status, as noted in Methods, does not necessarily indicate a state in conflict–countries with low policy and institutional capacity are also included in this group [34]. Comoros, for example, is considered a fragile state because of its low Country Policy and Institutional Assessment country rating, and has only 5% prevalence of recent IPV. Heterogeneity in this group of fragile states, from those affected by recent conflict vs. protracted conflict vs. impaired state governance may impact women’s economic opportunities, as well as their differential exposure to violence [50], suggesting that this measure may need further review in future research.

These findings help dissect the complex relationship between financial inclusion and recent IPV, though further research is needed for a full understanding of mechanisms, particularly given the contextual and normative factors on which these the relationship is conditioned [51, 52]. For example, the ability to use a conventional bank account generally necessitates some mobility, including the ability to physically go to a bank, something that is not possible for one in three women in low and middle income countries [16]. Given that IPV tends to be most prevalent in settings with restrictive gender norms, this presents an ongoing challenge to women’s economic engagement [53, 54]. While digital financial services such as mobile money accounts may mitigate some of these barriers, these digital accounts still require access to cell phones as well as financial literacy. This is an area for future growth, as currently, mobile accounts are only used by 3% of women globally (4% in low and middle income countries) [3].

Of course these findings must be interpreted in light of limitations inherent in any cross-sectional, ecological analysis, namely that causality cannot be inferred, and that results do not necessarily translate to the community or individual level. Data were collected in different years, though the year of IPV data collection was accounted for in current analyses. We were limited to measures that are publicly available, which do not fully assess all aspects of asset-based enablers of economic autonomy, gender norms or national context, and exclude, for example, measures of independent mobility. Nevertheless, these findings are suggestive and lend important insights that help to unpack the complex relationship between women’s financial inclusion and IPV.

Both preventing IPV and expanding financial inclusion have attained major prominence on the global development agenda. Encouragingly, our ecological results suggest that financial inclusion may be an important lever in reducing women’s risk of IPV, but both IPV and financial inclusion exist in the context of social, cultural and normative barriers and enablers. These barriers and enablers influence underlying mechanisms, as well as the country-level manifestations of these relationships. This means that we should not assume that financial inclusion is a universally positive component of development. Our findings underline the importance of shifting underlying norms and controlling behaviors which heighten the risk of violence, and suggest that efforts to promote women’s empowerment may be undermined in the absence of those changes. We add to the body of research suggesting that financial inclusion merits focus within the context of broader efforts to improve the status of women and reduce gender inequitable norms, and that this may offer opportunities to reduce women’s risk of violence at the hands of their partners.

## Supporting information

S1 Table Year of IPV and financial inclusion data collection for assessed countries. (DOCX)

S2 Table Descriptive statistics of assessed variables stratified by tertile of controlling behavior. (DOCX)
