# Supplementary material for: Financial inclusion and intimate partner violence: What does the evidence suggest?
Source: PLoS One. 2019 Oct 16;14(10):e0223721. doi: 10.1371/journal.pone.0223721 (PMC6795492; doi:10.1371/journal.pone.0223721)
Supplement: S1 Table — (DOCX) [file pone.0223721.s001.docx]

**S1 Table. Year of IPV and financial inclusion data collection for assessed countries.**

| Country | Recent physical or sexual IPV | Financial inclusion |
| --- | --- | --- |
| **High income** |  |  |
| Argentina | 2015^[[1]](#endnote-1)^ | 2014 |
| Australia | 2012 | 2011 |
| Austria | 2014 | 2014 |
| Belgium | 2014 | 2014 |
| Canada | 2011 | 2011 |
| Croatia | 2014 | 2014 |
| Cyprus | 2014 | 2014 |
| Czech Republic | 2014 | 2014 |
| Denmark | 2014 | 2014 |
| Estonia | 2014 | 2014 |
| Finland | 2014 | 2014 |
| France | 2014 | 2014 |
| Germany | 2014 | 2014 |
| Greece | 2014 | 2014 |
| Hungary | 2014 | 2014 |
| Ireland | 2014 | 2014 |
| Italy | 2014 | 2014 |
| Latvia | 2014 | 2014 |
| Lithuania | 2014 | 2014 |
| Luxembourg | 2014 | 2014 |
| Malta | 2014 | 2014 |
| Netherlands | 2014 | 2014 |
| Norway | 2008 | 2014 |
| Poland | 2014 | 2014 |
| Portugal | 2014 | 2014 |
| Singapore | 2013 | 2014 |
| Slovakia | 2014 | 2014 |
| Slovenia | 2014 | 2014 |
| Spain | 2014 | 2014 |
| Sweden | 2014 | 2014 |
| Trinidad and Tobago | 2018 | 2017 |
| United Kingdom of Great Britain and Northern Ireland | 2014 | 2014 |
| United States | 2010^[[2]](#endnote-2)^ | 2011 |
| **Central Asia and Southern Asia** |  |  |
| Afghanistan | 2015 | 2014 |
| Bangladesh | 2007 | 2011 |
| Bhutan | 2012 | 2014 |
| Bosnia and Herzegovina | 2013^[[3]](#endnote-3)^ | 2014 |
| India | 2016 | 2017 |
| Kyrgyzstan | 2012 | 2011 |
| Nepal | 2016 | 2017 |
| Pakistan | 2018^[[4]](#endnote-4)^ | 2017 |
| Russian Federation | 2011^[[5]](#endnote-5)^ | 2011 |
| Tajikistan | 2012 | 2011 |
| **Eastern Asia and South-Eastern Asia** |  |  |
| Indonesia | 2016 | 2017 |
| Cambodia | 2015 | 2014 |
| Lao People's Democratic Republic | 2014 | 2017 |
| Mongolia | 2017^[[6]](#endnote-6)^ | 2017 |
| Myanmar | 2016 | 2017 |
| Philippines | 2013 | 2014 |
| Vietnam | 2010 | 2011 |
| **Latin America and the Caribbean** |  |  |
| Bolivia (Plurinational State of) | 2008 | 2011 |
| Brazil | 2017^[[7]](#endnote-7)^ | 2017 |
| Chile | 2017^[[8]](#endnote-8)^ | 2017 |
| Colombia | 2015 | 2014 |
| Dominican Republic | 2013 | 2014 |
| Ecuador | 2011^[[9]](#endnote-9)^ | 2011 |
| El Salvador | 2008 | 2011 |
| Guatemala | 2015 | 2014 |
| Haiti | 2013 | 2014 |
| Honduras | 2012 | 2011 |
| Jamaica | 2008 | 2011 |
| Mexico | 2011 | 2011 |
| Nicaragua | 2012 | 2011 |
| Panama | 2009^[[10]](#endnote-10)^ | 2011 |
| Paraguay | 2008^[[11]](#endnote-11)^ | 2011 |
| Peru | 2016 | 2017 |
| Uruguay | 2013^[[12]](#endnote-12)^ | 2014 |
| **Northern America and Europe** |  |  |
| Bulgaria | 2014 | 2014 |
| Republic of Moldova | 2011 | 2011 |
| Romania | 2014 | 2014 |
| Ukraine | 2007 | 2011 |
| **Sub-Saharan Africa** |  |  |
| Angola | 2016 | 2014 |
| Burundi | 2017^[[13]](#endnote-13)^ | 2014 |
| Burkina Faso | 2010 | 2011 |
| Central African Republic | 2014 | 2011 |
| Côte d'Ivoire | 2012 | 2014 |
| Cameroon | 2014 | 2014 |
| Chad | 2015 | 2014 |
| Comoros | 2012 | 2011 |
| Democratic Republic of the Congo | 2014 | 2014 |
| Ethiopia | 2016 | 2017 |
| Gabon | 2012 | 2011 |
| Ghana | 2008 | 2011 |
| Guinea | 2011 | 2011 |
| Kenya | 2014 | 2014 |
| Lesotho | 2014^[[14]](#endnote-14)^ | 2011 |
| Liberia | 2007 | 2011 |
| Malawi | 2016 | 2017 |
| Mali | 2014 | 2014 |
| Mauritius | 2017^[[15]](#endnote-15)^ | 2017 |
| Mozambique | 2015 | 2017 |
| Namibia | 2013 | 2014 |
| Nigeria | 2013 | 2014 |
| Rwanda | 2015 | 2014 |
| Sierra Leone | 2013 | 2014 |
| South Africa | 2016^[[16]](#endnote-16)^ | 2017 |
| Togo | 2014 | 2014 |
| Uganda | 2016 | 2017 |
| United Republic of Tanzania | 2016 | 2017 |
| Zambia | 2014 | 2014 |
| Zimbabwe | 2015 | 2014 |
| **Western Asia and Northern Africa** |  |  |
| Albania | 2013^[[17]](#endnote-17)^ | 2014 |
| Algeria | 2006^[[18]](#endnote-18)^ | 2011 |
| Armenia | 2016 | 2017 |
| Azerbaijan | 2006 | 2011 |
| Egypt | 2014 | 2014 |
| Georgia | 2017 | 2017 |
| Jordan | 2012 | 2011 |
| Lebanon | 2016^[[19]](#endnote-19)^ | 2017 |
| Morocco | 2009^[[20]](#endnote-20)^ | 2017 |
| Tunisia | 2010^[[21]](#endnote-21)^ | 2014 |
| Turkey | 2015 | 2014 |

Countries are alphabetically grouped by region. Sources of IPV data are UN Women Global Database on Violence against Women unless otherwise noted. Financial inclusion data comes from the World Bank’s Global Findex Data.

1. Arias M. Primer Estudio Nacional sobre Violencias Contra la Mujer 2015: basado en la International Violence Against Women Survey (IVAWS). Buenos Aires: Ciudad Autonoma de Buenos Aires: Ediciones SAIJ; 2017. [↑](#endnote-ref-1)
2. Breiding M, Chen J, Black M. Intimate Partner Violence in the United States—2010. Atlanta, GA, USA: Centers for Disease Control and Prevention; 2014. [↑](#endnote-ref-2)
3. Bosnia and Herzegovina Ministry for Human Rights and Refugees. Framework Strategy for the Implementation of the Convention on Preventing and Combating Violence Against Women and Domestic Violence in Bosnia and Herzegovina for the Period 2015-2018. Bosnia and Herzegovina Ministry for Human Rights and Refugees; 2013. [↑](#endnote-ref-3)
4. National Institute of Population Studies (NIPS) [Pakistan] and ICF. Pakistan Demographic and Health Survey 2017-18. Islamabad, Pakistan and Rockville, Maryland, USA: NIPS and ICF; 2019. [↑](#endnote-ref-4)
5. Federal State Statistic Service (ROSSTAT), United Nations Population Fund (UNFPA), Division of Reproductive Health, CDC. Reproductive Health Survey Russia 2011: Executive Summary. Atlanta, GA, USA: Federal State Statistic Service (ROSSTAT), United Nations Population Fund (UNFPA), Division of Reproductive Health, CDC; 2012. [↑](#endnote-ref-5)
6. National Statistics Office of Mongolia, UN Population Fund. Breaking the silence for equality: 2017 National Study on Gender-based Violence in Mongolia. Ulaanbaatar, Mongolia: National Statistics Office of Mongolia and UN Population Fund; 2018. [↑](#endnote-ref-6)
7. Instituto de Pesquisa Data Senado. Violência doméstica e familiar contra a mulher. Brazil: Instituto de Pesquisa DataSenado, Observatório da Mulher contra a Violência; 2018. [↑](#endnote-ref-7)
8. Ministerio del Interior y Seguridad Pública Chile. Tercera encuesta nacional de violencia intrafamiliar contra la mujer y delitos sexuales: Presentación de resultados. Santiago, Chile: Subsecretaría de Prevencion del Delito, Ministerio del Interior y Seguridad Pública; 2017. [↑](#endnote-ref-8)
9. Encuesta Nacional de Relaciones Familiares y Violencia de Género contra las Mujeres 2011, Ecuador [Internet]. Secretaría Nacional de Planificación y Desarrollo (SENPLADES). 2011 [cited 15 July 2018]. Available from: <http://anda.inec.gob.ec/anda/index.php/catalog/94/related_materials>. [↑](#endnote-ref-9)
10. Richardson RGDL, García LM, Eric E, Chu V, Q AIM, Mojica FC, et al. Encuesta Nacional de Salud Sexual y Reproductiva (ENASSER) Panamá, 2009: Informe final. Panama: Instituto Conmemorativo Gorgas de Estudios de la Salud; 2011. [↑](#endnote-ref-10)
11. Paraguay Center for Population Studies (CEPEP). Encuesta Nacional de Demograf a y Salud Sexual y Reproductiva 2008. Asunción, Paraguay: Paraguay Center for Population Studies (CEPEP); 2009. [↑](#endnote-ref-11)
12. Encuesta Nacional de Prevalencia sobre Violencia Basada en Género y Generaciones 2013 [Internet]. Instituto Nacional de Estadística - Presidencia de la República, INMUJERES - MIDES. 2013 [cited 15 July 2018]. Available from: <http://www3.ine.gub.uy:82/anda4/index.php/catalog/681> [↑](#endnote-ref-12)
13. Ministère à la Présidence chargé de la Bonne Gouvernance et du Plan Burundi (MPBGP), Ministère de la Santé Publique et de la Lutte contre le Sida Burundi (MSPLS), Institut de Statistiques et d’Études Économiques du Burundi (ISTEEBU), ICF. Troisième Enquête Démographique et de Santé. Bujumbura, Burundi: ISTEEBU, MSPLS, et ICF; 2017.. [↑](#endnote-ref-13)
14. Chipatiso LM, Machisa M, Nyambo V, Chiramba K. The gender-based violence indicators study: Lesotho. Johannesburg, South Africa: Gender Links; 2014. [↑](#endnote-ref-14)
15. Sultan R. A Quantitative Assessment of Intimate Partner Violence and Associated Economic Costs in Mauritius. Reduit, Mauritius: University of Mauritius; 2017. [↑](#endnote-ref-15)
16. National Department of Health (NDoH), Statistics South Africa (Stats SA), South African Medical Research Council (SAMRC), ICF. South Africa Demographic and Health Survey 2016. Pretoria, South Africa and Rockville, Maryland, USA: NDoH, Stats SA, SAMRC and ICF; 2019. [↑](#endnote-ref-16)
17. Haarr R. Domestic violence in Albania: National population-based survey. Albania: INSTAT, UN ALbania, UNDP, Government of Sweden; 2013. [↑](#endnote-ref-17)
18. Ministère Délégué Chargé de la Famille et de la Condition Féminine. Stratégie Nationale de Lutte contre la Violence à l'égard des Femmes (2007-2011). Algeria: République Algérienne Démocratique et Populaire; 2009. [↑](#endnote-ref-18)
19. Mansour Z, Deeb M, Brandt L, Said R, Torossian L. Understanding masculinities: Results from the International Men and Gender Equality Survey (IMAGES) in Lebanon. Promundo, CRD, IMAGES MENA, UN WOMEN; 2017. [↑](#endnote-ref-19)
20. Royaume du Maroc: Haut - Commissariat au Plan. Enquête nationale sur la prévalence de la violence à l’égard des femmes au Maroc 2009. Morocco: Royaume du Maroc; 2009. [↑](#endnote-ref-20)
21. Projet de Coopération ONFP/AECID « Promotion de l’Equité de Genre et Prévention de la Violence à l’Egard des Femmes ». Enquête Nationale Sur la violence à l’égard des femmes en Tunisie: Rapport de l’enquête. Tunis, tunisia: ONFP/AECID; 2010. [↑](#endnote-ref-21)
