# Supplementary material for: Financial inclusion and intimate partner violence: What does the evidence suggest?
Source: PLoS One. 2019 Oct 16;14(10):e0223721. doi: 10.1371/journal.pone.0223721 (PMC6795492; doi:10.1371/journal.pone.0223721)
Supplement: S2 Table — (DOCX) [file pone.0223721.s002.docx]

**S2 Table. Descriptive statistics of assessed variables stratified by tertile of controlling behavior.**

|  | Lowest tertile (n=15)^1^ | Middle tertile (n=16)^2^ | Highest tertile (n = 15)^3^ |
| --- | --- | --- | --- |
|  | Median (range) | Median (range) | Median (range) |
| Recent IPV (%) | 15.5 (3.5-31.6) | 14.4 (4.9-46.1) | 26.7 (9.8-36.8) |
| Financial inclusion (%) | 34.7 (6.7-76.6) | 24.7 (3.8-71.1) | 14.7 (2.1-52.7) |
| Financial inclusion gender gap (%) | 8.4 (-13.5 - 27.6) | 7.7 (-3.8 – 20.3) | 6.4 (-0.4 – 16.3) |
| ***Asset-based enablers of economic autonomy*** | | | |
| Female employment (%) | 54.4 (24.0-92.7) | 61.5 (42.9-82.0) | 65.7 (13.5-83.0) |
| Cash earnings (%) | 72.6 (16.9-94.7) | 69.0 (35.5-95.9) | 54.4 (27.5-88.0) |
| Cell phone use (%) | 67.3 (25.9-92.4) | 74.3 (43.1-89.1) | 68.5 (45.7-93.0) |
| Female education (years) | 4.3 (1.4-10.6) | 6.2 (2.0-10.9) | 5.5 (2.5-10.7) |
| ***Gender norms*** | | | |
| Inequitable employment norms (%) | 17.5 (6.0-73.0) | 11.0 (7.0-51.0) | 22.0 (11.0-48.0) |
| Decision-making over own earnings (%) | 90.4 (81.7-98.2) | 91.7 (65.0-98.3) | 91.0 (69.5-95.5) |
| Controlling behavior (%) | 44.9 (27.2-56.7) | 65.2 (60.5-68.9) | 78.0 (71.4-86.4) |
| Wife-beating justified (%) | 41.1 (5.5-76.3) | 35.7 (2.3-80.2) | 49.0 (16.3-74.8) |
| ***National context*** | | | |
| HDI | 0.57 (0.41-0.75) | 0.54 (0.38-0.75) | 0.55 (0.39-0.73) |
| Fragile state^4^ |  |  |  |
| No | 11 (73.3) | 10 (62.5) | 11 (73.3) |
| Yes | 4 (26.7) | 6 (37.5) | 4 (26.7) |

^1^ Armenia, Burundi, Cambodia, Ethiopia, Guatemala, India, Mali, Mozambique, Myanmar, Namibia, Nepal, Pakistan, Philippines, Rwanda and South Africa.

^2^ Afghanistan, Angola, Burkina Faso, Chad, Comoros, Côte d’Ivoire, Dominican Republic, Ghana, Honduras, Kenya, Nigeria, Peru, Republic of Moldova, Togo, Ukraine and Zimbabwe.

^3^ Azerbaijan, Cameroon, Democratic Republic of the Congo, Egypt, Gabon, Haiti, Jordan, Kyrgyzstan, Liberia, Malawi, Sierra Leone, Tajikistan, Uganda, United Republic of Tanzania and Zambia.

^4^ N (%)
